# Beyond Plant Microbiome Composition: Exploiting Microbial Functions and Plant Traits via Integrated Approaches

**DOI:** 10.3389/fbioe.2020.00896

**Published:** 2020-08-07

**Authors:** Chunxu Song, Feng Zhu, Víctor J. Carrión, Viviane Cordovez

**Affiliations:** ^1^College of Resources and Environmental Sciences, China Agricultural University, Beijing, China; ^2^National Academy of Agriculture Green Development, China Agricultural University, Beijing, China; ^3^Key Laboratory of Plant-Soil Interactions, Ministry of Education, China Agricultural University, Beijing, China; ^4^Key Laboratory of Agricultural Water Resources, Hebei Key Laboratory of Soil Ecology, Center for Agricultural Resources Research, Institute of Genetic and Developmental Biology, Chinese Academy of Sciences, Shijiazhuang, China; ^5^Institute of Biology, Leiden University, Leiden, Netherlands; ^6^Department of Microbial Ecology, Netherlands Institute of Ecology (NIOO-KNAW), Wageningen, Netherlands

**Keywords:** microbiome engineering, host-mediated selection, agricultural practices, interactions, plant growth, plant health

## Abstract

Plants recruit specific microorganisms to live inside and outside their roots that provide essential functions for plant growth and health. The study of the microbial communities living in close association with plants helps in understanding the mechanisms involved in these beneficial interactions. Currently, most of the research in this field has been focusing on the description of the taxonomic composition of the microbiome. Therefore, a focus on the plant-associated microbiome functions is pivotal for the development of novel agricultural practices which, in turn, will increase plant fitness. Recent advances in microbiome research using model plant species started to shed light on the functions of specific microorganisms and the underlying mechanisms of plant–microbial interaction. Here, we review (1) microbiome-mediated functions associated with plant growth and protection, (2) insights from native and agricultural habitats that can be used to improve soil health and crop productivity, (3) current -omics and new approaches for studying the plant microbiome, and (4) challenges and future perspectives for exploiting the plant microbiome for beneficial outcomes. We posit that integrated approaches will help in translating fundamental knowledge into agricultural practices.

## Introduction

**FIGURE 1:**
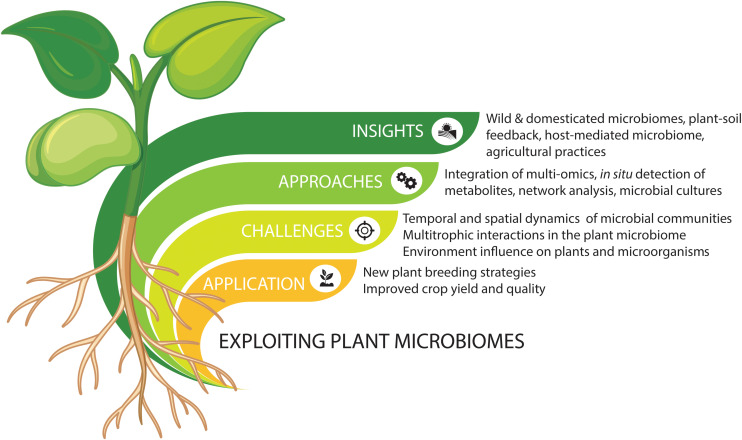
Summary of integrated approaches to explore and exploit plant microbiomes for new agricultural strategies. Vectors designed by Brgfx (Freepik).

The plant microbiome comprises a plethora of beneficial, commensal, and pathogenic microorganisms that play important roles in plant growth and health (Vorholt, 2012; Mendes et al., 2013; Philippot et al., 2013; Raaijmakers and Mazzola, 2016; Lemanceau et al., 2017). Advances in high-throughput sequencing have been increasing our understanding of microbial community composition and taxa-specific distributions. However, understanding the mechanisms underlying the interactions between plants and their microbiomes requires a focus on linking microbiome structure to functions. Metagenomic and metatranscriptomic analyses have started unraveling the functions of plant-associated microbiomes in both natural and agricultural systems. The next challenge is to develop new methods for effective application and manipulation in agricultural settings. Here, we discuss some of the beneficial microbiome-mediated functions for plant growth and protection and highlight some future strategies for engineering the plant microbiome (Figure 1).

## Microbiome-Mediated Functions for Plant Protection

Microorganisms can directly impact plant growth and health by producing phytohormones, improving nutrient acquisition and phosphate solubilization, and indirectly by activating the plant immune responses or competing and inhibiting the growth of plant pathogens, for example, via the production of antibiotics, fungal cell wall–degrading enzymes, and siderophore activity (Gamalero and Glick, 2011; Berendsen et al., 2012; Gu et al., 2020). An increasing number of studies have shown that, in most cases, not a single microbe but rather a consortium of microorganisms is responsible for the beneficial effects on plants. For example, three bacterial genera, *Microbacterium*, *Stenotrophomonas*, and *Xanthomonas*, were found to be enriched in the rhizosphere of *Arabidopsis thaliana* upon foliar defense activation by the downy mildew pathogen *Hyaloperonospora arabidopsidis* (Berendsen et al., 2018). Further investigation revealed that separately these bacteria do not impact plant growth and health, but when acting in a consortium, induced systemic resistance and plant growth promotion could be achieved. In another study, a consortium of endophytes, including the fungi *Rhodotorula graminis*, and the bacteria *Burkholderia vietnamiensis*, *Rhizobium tropici*, *Acinetobacter calcoaceticus*, *Rahnella* sp., *Burkholderia* sp., *Sphingomonas yanoikuyae*, *Pseudomonas* sp., and *Curtobacterium* sp., enhanced drought stress tolerance of poplar plants, thus suggesting a potential role in plant stress response modulation, for example, reduction of damage by reactive oxygen species (Khan et al., 2016). Members of the endosphere and rhizosphere microbiome have also been shown to suppress plant diseases, such as the take-all disease caused by the fungal pathogen *Gaeumannomyces graminis* (Durán et al., 2017, 2018) and the damping-off disease caused by *Rhizoctonia solani* (Mendes et al., 2011; Carrión et al., 2018). Furthermore, specific members in the rhizosphere of tomato plants provided resistance to *Ralstonia solanacearum*, a bacterial pathogen that causes wilt disease (Kwak et al., 2018). Transplantation of the rhizosphere microbiome from resistant to susceptible plants provided disease suppression. A highly abundant Flavobacteria metagenome-assembled genome (MAG) was detected in the rhizosphere of the resistant plants. Using a culture-dependent approach, *Flavobacterium* sp. TRM1 was isolated and the mediated suppression against wilt disease was confirmed. A study on the endosphere microbiome of sugar beet plants grown in a soil suppressive to *R. solani* revealed enrichment of Bacteroidetes, particularly those belonging to the *Chitinophaga* and *Flavobacterium* genera which synergistically suppressed the pathogen. Metagenomic analysis of this suppressive soil identified a biosynthetic gene cluster (BGC) encoding for a polyketide synthase-non-ribosomal peptide synthetase enzyme in the genome of the isolated *Flavobacterium*, and site-directed mutagenesis in the BGC confirmed its involvement in disease suppression (Carrión et al., 2019).

## Insights From Native and Agricultural Habitats on Microbiome Assembly

Studies on the microbiome of plants with natural resistance to (a)biotic stresses or plants growing in unfavorable environments can provide insights into microbial and plant traits that allow them to withstand such conditions. Wild plants display higher tolerance to abiotic (e.g., drought and salinity) and biotic (e.g., pests and pathogens) stresses when compared with domesticated plants. These wild relatives have been exploited by plant breeders as sources of resistance genes and specific traits (Hajjar and Hodgkin, 2007; Turcotte et al., 2014; Zhang et al., 2017). The development of crop varieties with higher yields and other desirable agronomic traits via domestication and breeding programs have significantly altered root architecture and traits, as well as shrinking the plant genetic diversity (Micallef et al., 2009; Pérez-Jaramillo et al., 2016; Cordovez et al., 2019). An increasing number of studies has shown differences in microbiome composition and functions in the rhizosphere of modern and wild plants, as well as of plants grown in native and agricultural soils (Zachow et al., 2014; Bulgarelli et al., 2015; Coleman-Derr et al., 2016; Pérez-Jaramillo et al., 2018, 2019; Brisson et al., 2019). Particularly, members of Bacteroidetes were found to be highly abundant in wild relatives as compared with modern sugar beet, common bean, and barley plants (Pérez-Jaramillo et al., 2017, 2018).

Agricultural practices also impact the plant microbiome composition and functions (Hartman et al., 2017, 2018) with negative and positive consequences on plant growth. Cropping systems, such as intercropping and no-tilling organic farming, increased microbial community diversity, crop yield, and soil organic carbon levels (Mader, 2002; Debenport et al., 2015; Reganold and Wachter, 2016; Wang et al., 2017). On the other hand, monoculture or short rotations of crops more rapidly deplete soil nutrients and increase plant species–specific soil pathogens and root herbivores, thus resulting in yield decrease (Bennett et al., 2012; Hilton et al., 2013; Santhanam et al., 2015; McDonald and Stukenbrock, 2016). Exploration of the microbiome of wild plants in their native habitats combined with the knowledge on how the different agricultural practices impact microbial communities is an interesting aspect that is used to unravel and, ultimately, reinstate beneficial associations that may have been undermined throughout plant domestication. Besides, a key aspect to be explored in breeding programs will be the identification of plant genes involved in the recruitment of beneficial microorganisms under different agricultural practices. These innovative approaches will enable plants to select and maintain beneficial microbiomes (Schlatter et al., 2017; Compant et al., 2019).

Plants alter the (a)biotic properties of the environment where they grow via the production of root exudates. To some extent, these properties will also impact the growth and health of the current and future plant generations (Kulmatiski and Kardol, 2008; van der Putten et al., 2013; Pineda et al., 2020). This process is known as plant–soil feedbacks (PSFs) and often relates to changes in soil nutrient availability, or the antagonistic and beneficial interactions between plant and soil microbial communities (Bennett and Klironomos, 2019). For example, benzoxazinoids, a defensive root exudate released by cereals, alter root-associated microbiome, decrease plant growth, and suppress herbivore performance in the next plant generation as well as the root-associated microbiome (Hu et al., 2018). Furthermore, PSFs are exploited in the form of crop rotation that can provide optimal soil legacy for crop yield, quality, and environmental sustainability (Mariotte et al., 2018; Heinen et al., 2020).

## Current-Omics and Other Approaches for Studying the Plant Microbiome

To study the complexity of genetic, microbial, and metabolic factors that impact the plant microbiome, comprehensive systems biology approaches are needed (Rodriguez et al., 2019). Metagenomics and metatranscriptomics allow us to go beyond the description of taxonomic changes in microbial taxa abundances and provide further information on microbial functions. In addition, proteomics and metabolomics, that is, the analysis of the intermediate and final products of genes, provide evidence of functional proteins and metabolites. Metagenomic coupled with metaproteomic approaches highlighted proteins involved in methane production in the rice rhizosphere (Knief et al., 2012).

Studies of the metabolites produced by plant-associated microbiomes *in situ* are still limited and complex owing to the dynamics and heterogeneity of the soil environment. Soil metabolites are composed of both plant and microbial secreted molecules and their production is influenced by the soil properties and by the interactions of soil microorganisms with host plants (Pétriacq et al., 2017; Mhlongo et al., 2018; Hayden et al., 2019; Jacoby and Kopriva, 2019; Nguyen et al., 2020). Moreover, recent studies have also been using new approaches to study soil metabolites. For example, liquid chromatography–mass spectrometry (LC-MS) and proton nuclear magnetic resonance spectroscopy (^1^H NMR) revealed a higher abundance of sugar-derived molecules in suppressive soils than non-suppressive soils, and the latter also contained higher amounts of lipids and terpenes (Hayden et al., 2019). It is important noticing that traditional mass spectrometry–based metabolomic analyses require high input of time in sample preparation, extraction, and purification. In addition, ambient ionization allows the generation of metabolite ions in normal atmospheric conditions without sample preparation or extraction, whereas mass spectrometry imaging (MSI) allows the visualization of the spatial distribution of metabolites in real time (Watrous and Dorrestein, 2011; Cameron and Takáts, 2018). MSI has been successfully applied to study microbial interactions, such as bacteria–protist interaction (Song et al., 2015), bacteria–fungi interaction (de Bruijn et al., 2015), and bacteria–plant interaction (Debois et al., 2014). This technique allows not only to investigate the metabolites of microbial communities *in situ* but also the spatial resolution ranging from nanometers to millimeters (Watrous and Dorrestein, 2011).

For an analytical aspect, network analysis of microbial communities has shown to be a promising first step to identify potential microbe–microbe interactions and explore their dynamics (Faust and Raes, 2012). For example, increased network size and complexity were found in the rhizosphere microbiome of *Avena fatua* plants, whereas networks in the surrounding soil remained relatively static and simple over time (Shi et al., 2016). Increased network complexity suggests a greater potential for interactions and niche-partitioning in the rhizosphere. Network analysis of the rhizosphere of common beans which were resistant and susceptible to the fungal pathogen *Fusarium oxysporum* revealed a more complex and interconnected bacterial community for the resistant cultivar as compared with the susceptible one (Mendes et al., 2018b). In the community of resistant plants, *Paenibacillus* was found to be a keystone genus (Mendes et al., 2018a). Moreover, co-occurrence network analyses have also demonstrated that interactions between co-existing organisms impact how microbial communities respond to changes in their environment (de Vries et al., 2018). For example, under drought stress, fungal communities were shown to be more stable than bacterial communities. The drought was also found to have a prolonged impact on bacterial communities and their networks via changes in vegetation composition (de Vries et al., 2018). These studies demonstrate that network inference can provide perspectives on microbial communities beyond those of species richness and composition (Shi et al., 2016).

## Current Challenges and Future Perspectives for Exploiting the Plant Microbiome

### Moving Beyond Bacterial and Fungal Communities

The plant microbiome encompasses distinct microbial groups, such as bacteria, fungi, viruses, algae, and protozoa. Currently, the majority of microbiome studies had focused on bacterial and fungal communities. However, plant interactions with other members of the microbiome as well as interactions across these microorganisms determine the overall diversity and functioning. Recent studies have shown that protists play important roles in the soil microbiome and in plant health (Thakur and Geisen, 2019). For example, Xiong et al. (2020) found that the pathogen dynamics is best predicted by protists, which were found to be negatively correlated with pathogen abundance during the growth of tomato plants. By directly feeding on the pathogen or indirectly by inducing shifts in the taxonomic and functional composition of bacteria via predation, protists might provide plant protection. Also, bacteriophages have shown to play important roles in the rhizosphere of tomato plants. Different phage combinations decreased the incidence of tomato disease *Ralstonia solanacearum* infection by up to 80% (Wang et al., 2019). The effects of phages on the pathogen indirectly altered the bacterial community, enriching for taxa (*Acinetobacter*, *Bacillus*, *Comamonas*, *Ensifer*, and *Rhodococcus*) that antagonize the pathogen.

### Expanding Microbial Cultures and Validating Their Functions

A number of studies have validated the role of specific microbial synthetic communities (SynComs) using culturable microbial consortia on plant drought tolerance (Khan et al., 2016), soil disease suppression (Carrión et al., 2019), and plant growth promotion (Zhang et al., 2019). To date, different strategies have been developed to design SynComs and deployed them in soil–plant system to achieve a desirable function. For example, Vorholt et al. (2017) proposed reductionist strategies for constructing SynComs based on phylogeny, classification, interaction networks, or specific functions. Oyserman et al. (2018) proposed microbiome-associated phenotypes (MAPs) for developing “modular microbiomes,” that is, SynComs that are engineered cooperatively with the host genotype to confer different but mutually compatible MAPs to a single host or host population. This host-mediated microbiome selection approach also allows the identification of both host and microbial traits and genes that co-evolved.

In addition, the identification of substrate preferences might contribute to the culture of microorganisms which can be further used as SynComs. For example, a study coupling genomics and exometabolomics showed that chemical succession in the rhizosphere interacts with metabolite substrate preferences by microorganisms that are predictable from genome sequences (Zhalnina et al., 2018). This approach revealed that rhizosphere-enriched and rhizosphere-depleted strains exhibit the likelihood to catabolize aromatic organic acids and nucleotides, respectively, to colonize specific niches. These findings demonstrate the chemical cues governing microbial community assembly in the rhizosphere and provide an attractive direction for promoting the growth of specific members of the microbiome which can have beneficial effects for plants.

### Understanding the Interactions Between Plant Genotype, Microbiome, and Environment

The current challenge for exploiting and applying microbiome-mediated functions that positively influence plant growth and protection across diverse environments rely on critical aspects of context dependency. In other words, these beneficial effects are largely dependent on plant genotypes, microbial interactions, soil types, management practices as well as interactions among these factors (Fierer and Jackson, 2006; van Elsas et al., 2012; Rodrigues et al., 2013; Hunter, 2016; Busby et al., 2017; Soman et al., 2017; Schmidt et al., 2019). Although engineering microbiomes that provide beneficial effects globally remains a challenge, understanding the interaction among plant genotype, microbiome, and environment will contribute to define microbial consortia that can persist in a variety of heterogeneous ecosystems (Agler et al., 2016; Busby et al., 2017). Also, the abundance and activity of microorganisms can be influenced by single members of the communities. A study using SynComs with seven representative bacterial strains from the three most abundant phyla in maize roots showed that the removal of one strain resulted in the collapse of the community (Niu et al., 2017). These findings demonstrate the importance of individual members of the microbiome for specific functions.

### Exploiting Host-Mediated Selection of Microbiome Members and Functions

In agriculture, microbial inoculation of single strains with beneficial traits has been used for disease management and promoting yield gain (Berg, 2009; Lugtenberg and Kamilova, 2009; Gamalero and Glick, 2011; Chaparro et al., 2012). However, the application under field conditions has been limited. Establishing the strain at the right time and at the right concentration, often under adverse environmental conditions, are limiting factors for the successful colonization of the applied strain in soil (Sessitsch et al., 2019). Stable populations of beneficial microorganisms are selectively recruited and maintained in the rhizosphere by the plant via the exudation of carbon-rich compounds into the rhizosphere (Doornbos et al., 2012; Vives-Peris et al., 2020). Thus, a strategy for engineering beneficial microbiomes is to equip plants to recruit the beneficial members of the microbial community, for example, throughout plant breeding programs. New strategies for selection of microbiomes, such as host-mediated microbiome selection (Mueller and Sachs, 2015), have been proposed and are based on the hypothesis that plants have evolved to selectively recruit beneficial microorganisms which can be subsequently transmitted to the next generation of plants.

## Conclusion

Our understanding of the plant microbiome has vastly increased in the past decade. Integrated approaches, such as different multi-omics and microbiome engineering strategies, have greatly contributed to a better understanding of the organization and dynamics of plant-associated microbial communities. In addition, plant–soil feedbacks opened a new avenue for improvement of agricultural practices using knowledge obtained from natural ecosystems. Plant breeding programs have traditionally focused on exploring genetic variability of the crops for higher productivity and stress resistance, often neglecting the importance of beneficial interactions between microorganisms and plants. Therefore, future strategies for plant breeding should take plant microbial symbionts as life-long bodyguards into consideration. These proposed integrated approaches will provide solutions for exploring and exploiting plant–microbiome interactions for improving the sustainability and productivity of global agriculture.

## Author Contributions

CS, FZ, VJC, and VC conceived and wrote the manuscript. All authors read and approved the final version of the article.

## Conflict of Interest

The authors declare that the research was conducted in the absence of any commercial or financial relationships that could be construed as a potential conflict of interest.
